# Higher systemic vascular resistance in individuals with a family history of hypertension

**DOI:** 10.1097/HJH.0000000000004133

**Published:** 2025-09-26

**Authors:** Emmi K.H. Värri, Johanna Tietäväinen, Lauri J. Suojanen, Manoj Kumar Choudhary, Jukka Mustonen, Jenni K. Koskela, Ilkka H. Pörsti

**Affiliations:** a Faculty of Medicine and Health Technology, Tampere University; b Department of Internal Medicine, Tampere University Hospital, Tampere, Finland

**Keywords:** blood pressure, family history, haemodynamics, hypertension

## Abstract

**Objective:**

To compare blood pressure (BP) and cardiovascular function between individuals with and without a family history of hypertension in a first-degree relative.

**Methods:**

The haemodynamics of participants with (*n* = 437) and without (*n* = 274) a family history of hypertension were recorded using continuous tonometric pulse wave analysis and whole-body impedance cardiography during passive head-up tilt.

**Results:**

The group with a family history of hypertension had a higher percentage of antihypertensive medication users (15.6 vs. 8%, *P* = 0.003) and higher office BP (143/91 vs. 140/87 mmHg, *P* < 0.05) than the group without hypertensive first-degree relatives. The proportion of men (51.9 vs. 55.1%) and the mean age (48.2 vs. 48.1 years) were similar in both groups. During head-up tilt, participants with a family history of hypertension consistently had 5/4 mmHg (systolic/diastolic) higher radial and aortic BP (*P* < 0.001 for all comparisons), a shorter aortic reflection time (−2.4 ms, *P* = 0.017), and a higher systemic vascular resistance (SVR) index (180 dyn s/cm^5^ m^2^, *P* < 0.001) than participants without hypertensive first-degree relatives. Central forward wave amplitude, pulse pressure, augmentation pressure, augmentation index, heart rate, cardiac output, and pulse wave velocity did not differ between the study groups. All haemodynamic variables changed significantly in response to head-up tilt with no differences between the two study groups.

**Conclusion:**

Participants with a family history of hypertension were characterized by elevated central and peripheral BP probably due to higher SVR, whereas the stiffness of large arteries was not higher. These findings highlight the role of SVR in the pathogenesis of primary hypertension.

## INTRODUCTION

High blood pressure (BP) is one of the major risk factors for cardiovascular disease worldwide [1]. The mechanisms leading to the development of essential hypertension are complex, with both genetic and environmental factors playing a role. Family history of hypertension is an acknowledged risk factor for high BP, and the prevalence of hypertension is higher in individuals with hypertensive family members [2,3]. Also, the offspring of hypertensive parents exhibit higher BP than descendants of normotensive participants, even prior to the development of manifest hypertension [4,5].

BP is determined by cardiac output, systemic vascular resistance (SVR), and arterial stiffness [6]. Large artery stiffness is a well established cardiovascular risk factor and an independent predictor of future hypertension [7,8]. The current evidence concerning SVR as a predictor of future hypertension is limited. A recent study of 1293 adults aged 30–45 years concluded that SVR predicted incident hypertension over a 4-year follow-up; however, this interpretation may warrant some caution [9].

Several studies exhibit alterations in cardiovascular function even before the development of high BP when comparing participants without hypertensive first-degree relatives to those with a family history of hypertension. However, the evidence is somewhat conflicting. The reported alterations include lower baroreflex sensitivity [10], higher forward wave amplitude (FWA), higher augmentation index (AIx), and higher arterial stiffness as measured by pulse wave velocity (PWV) [11,12]. These changes may already be present at a young age [13]. Additionally, sex may play a role, with men being more frequently affected than women [14,15]. However, some studies have found that arterial stiffness was not higher in young adults with a family history of hypertension, although their BP was higher than in controls [16,17]. Buus *et al.* reported higher AIx and left ventricular mass index in individuals with parental hypertension, while vascular resistance and arterial stiffness were similar to those in participants without hypertensive parents [18]. They discussed that higher cardiac output was possibly the factor that elevated AIx [18]. In a rather small study comprising 44 young males, forearm vascular resistance, measured using strain-gauge plethysmography, was higher in participants with a positive family history of hypertension [19].

In the present study, we compared cardiovascular function in participants with and without hypertensive first-degree family members. As posture can individually influence BP [20,21], and an exaggerated SBP response to standing is an independent predictor of adverse cardiovascular events [22], the present recordings were performed in both the supine position and during passive head–up tilt.

## METHODS

### Study population

The study consisted of a total of 711 individuals voluntarily participating in our study of noninvasive haemodynamics during years 2006–2018 (clinical trial registration NCT01742702). The participants were recruited through newspaper announcements and notices in Tampere University Hospital, University of Tampere, Varala Sports Institute, and local occupational healthcare [23–25]. We included participants with a hypertensive first-degree relative (*n* = 437) and controls without family history of hypertension (*n* = 274). Information about family history was obtained via a questionnaire, and the family member in question could be a parent, sibling, or child. The study complies with the Declaration of Helsinki and has been approved by the Tampere University Hospital Ethics Committee (code R06086 M).

### Haemodynamic recordings

A comprehensive haemodynamic recording protocol was carried out in a laboratory setting [21,23,24]. Cigarette smoking, heavy meals, and caffeine were prohibited for 4 h, and alcohol use for 24 h prior to the recordings. Those participants taking BP-lowering agents were advised to continue their medications normally. Participants were positioned on a tilt table in the supine position, with their left arm fixed to a support at a 90° angle, keeping the wrist at heart level both in the supine position and during head-up tilt. A tonometric sensor for pulse wave analysis was fixed to the left wrist (Colin BP-508T, Colin Medical Instruments Corp., USA), and an oscillometric brachial cuff was placed on the right upper arm for calibration. Electrodes for impedance cardiography were placed on the body surface. The protocol consisted of continuous 5-min recordings, both in the supine position and during passive head-up tilt to 60–65°. The mean value of each minute was calculated and used in the analyses. The tonometric signal was calibrated every 2.5 min with a contralateral brachial BP measurement [21,23,24].

Radial BP and pulse waveform were recorded using the tonometric sensor, and aortic BP was derived on-line with the SphygmoCor pulse wave monitoring system (SphygmoCor PWMx, AtCor medical, Australia) using a validated generalized transfer function [26]. Augmentation pressure, AIx, aortic pulse pressure, mean arterial pressure, and aortic reflection time were determined from the pulse waveform by the SphygmoCor software [21,23–25]. FWA was calculated as central pulse pressure minus augmentation pressure, and pulse pressure amplification as radial pulse pressure/aortic pulse pressure [27].

Whole-body impedance cardiography (CircMon, JR Medical Ltd., Tallinn, Estonia) was used to record beat-to-beat R-R intervals, PWV and extracellular water volume, and the following variables, which were normalized to body surface area: stroke index, cardiac index, and SVR index [21,23–25,28,29]. Previously, whole-body impedance cardiography has been demonstrated to provide reasonably accurate, reliable, and repeatable measurements of cardiac output and stroke volume, showing good agreement with thermodilution and direct oxygen Fick method across various clinical settings, including during head-up tilt and after coronary artery bypass surgery [28–31]. In addition, stroke volume measurements using whole-body impedance cardiography and three-dimensional ultrasound show good agreement during head-up tilt [32]. Using the radial tonometric BP signal, SVR index was calculated by subtracting assumed normal central venous pressure (4 mmHg) from mean arterial pressure and dividing the value by cardiac output [29].

### Laboratory analyses

Blood samples were collected after ~12 h of fasting. Plasma electrolytes, creatinine, cystatin C, C–reactive protein, glucose, and lipid determinations were carried out using Cobas Integra 700/800 (F. Hoffmann-LaRoche Ltd., Basel, Switzerland) or Cobas 6000, module c501 (Roche Diagnostics, Basel, Switzerland) and blood cell count by ADVIA 120 or 2120 (Bayer Healthcare, Tarrytown, New York, USA). Estimated glomerular filtration rate was calculated using the CKD-EPI formula [33]. Standard 12-lead electrocardiograms were registered and the Cornell voltage-duration product was calculated [34].

### Statistical analyses

The normality of variable distribution was tested using Levene's test. The demographic and laboratory data in the groups were compared using the chi-square test, *t* test, or Mann–Whitney *U* test, as appropriate. Haemodynamic differences between the groups in supine and upright positions were examined using generalized estimating equations. This method allowed for the analysis of repeated measurements of haemodynamic variables to assess the influences of group, posture, and their interaction on the variable of interest. A linear scale response was applied, and the autoregressive option was chosen for the correlation matrix, as successive serial measures of haemodynamics in individual participants are autocorrelated. As the participants showed differences in the percentage of antihypertensive medication users (Table 1) and plasma concentrations of low-density lipoprotein cholesterol and calcium (Table 2), the analyses were adjusted for these differences by using the variables as covariates. The PWV analyses were additionally adjusted for mean aortic pressure according to expert consensus [35].

**TABLE 1:** Demographics, clinical characteristics, and laboratory results of participants according to negative or positive family history of hypertension in first-degree relatives

	History of hypertension in first-degree relatives		
Variable	Absent (*n* = 274)	Present (*n* = 437)	*P* value
Male proportion (%)	55.1	51.9	0.440
Age (years)	48.1 (12.7)	48.2 (11.5)	0.714
Women: postmenopausal participants (%)	31.7	38.1	0.286
Current smokers (%)	13.9	12.1	0.491
Previous smokers (%)	29.3	32.0	0.454
Alcohol use (standard drinks/week)	2.8 [0.3, 6.0]	3.0 [1.0, 7.0]	0.291
Bouts of exercise (*n*/week)	3 [2, 4]	3 [2, 4]	0.783
Body mass index (kg/m^2^)	27.2 (4.7)	27.1 (4.5)	0.785
Extracellular water volume (l)	13.2 (1.8)	12.9 (2.0)	0.098
Extracellular water balance	1.01 (0.10)	1.00 (0.11)	0.557
Dyslipidaemia (%)	26.1	36.9	**0.010**
Diabetes (%)	0.7	0.7	1.000
Antihypertensive medication users (%)	8.0	15.6	**0.003**
ACE inhibitor (*n*/%)	10/3.7%	21/4.8%	0.463
Angiotensin receptor blocker (*n*/%)	2/0.7%	26/6.0%	**<0.001**
Beta blocker (*n*/%)	13/4.8%	31/7.1%	0.139
Alpha and beta blocker (*n*/%)	0/0%	3/0.7%	0.169
Calcium channel blocker (*n*/%)	9/3.3%	26/6.0%	0.110
Thiazide diuretic (*n*/%)	5/1.8%	28/6.4%	**0.005**
Statin users (*n*/%)	11/4.0%	31/7.1%	0.090
Seated brachial office BP (mmHg)^a^	140 (22)/87 (12)	143 (20)^a^/91 (11)^a^	0.041/<0.001
Supine brachial laboratory BP (mmHg)^b^	130 (18)/79 (12)	135 (18)^a^/83 (11)^a^	<0.001/<0.001

Results shown as mean (standard deviation) or median [25th, 75th percentile]. ACE, angiotensin-converting enzyme; BP, blood pressure.

a Measurement by physician.

b Measurement by research nurse.

**TABLE 2:** Fasting blood and plasma biochemistry results of participants according to negative or positive family history of hypertension in first-degree relatives

	History of hypertension in first-degree relatives		
Variable	Absent (*n* = 274)	Present (*n* = 437)	*P* value
Haemoglobin (g/l)	144 (12)	144 (12)	0.470
C-reactive protein (mg/l)	1.0 [0.5, 2.3]	0.9 [0.5, 1.8]	0.094
Creatinine (μmol/l)	75 (14)	74 (14)	0.444
Cystatin C (mg/l)	0.87 (0.17)	0.86 (0.16)	0.382
Estimated GFR (ml/min/1.73 m^2^)	96 (16)	97 (16)	0.537
Uric acid (μmol/l)	313 (78)	310 (79)	0.591
Sodium (mmol/l)	140.0 (2.0)	140.0 (2.1)	0.245
Potassium (mmol/l)	3.83 (0.29)	3.78 (0.31)	0.180
Renin activity (ng Ang I/ml/h)	0.8 [0.4, 1.4]	0.8 [0.4, 1.5]	0.695
Aldosterone (pmol/l)	436 [326, 606]	445 [330, 602]	0.994
Aldosterone to renin ratio	597 [350, 885]	591 [327, 833]	0.629
Total cholesterol (mmol/l)	5.0 (1.0)	5.2 (1.0)	**0.017**
Triglycerides (mmol/l)	1.0 [0.7, 1.5]	1.1 [0.8, 1.6]	0.064
HDL cholesterol (mmol/l)	1.5 (0.4)	1.6 (0.5)	0.761
LDL cholesterol (mmol/l)	3.0 (1.0)	3.2 (1.0)	**0.032**
Glucose (mmol/l)	5.5 (0.7)	5.5 (0.6)	0.445
25(OH)D_3_ (nmol/l)	66 [48, 91]	66 [49, 87]	0.697
1,25(OH)_2_D_3_ (pmol/l)	101 [82, 124]	104 [83, 128]	0.648
Parathyroid hormone (pmol/l)	4.2 [3.4, 5.4]	4.4 [3.4, 5.4]	0.693
Calcium (mmol/l)	2.30 (0.10)	2.32 (0.11)	**0.036**
Phosphate (mmol/l)	0.96 (0.17)	0.96 (0.16)	0.675
24-h Na^+^ excretion (mmol)	159 (64)	156 (61)	0.643
24-h K^+^ excretion (mmol)	85 (29)	83 (28)	0.492
Nocturnal albumin excretion (μg/min)	4 [3, 6]	4 [3, 6]	0.342
Cornell voltage-duration product	1694 (570)	1773 (594)	0.108

Results shown as mean (standard deviation) or median [25th and 75th percentile]. Numbers of observations: *n* = 271–273 for negative and 430–435 for positive family history, except for 1,25(OH)_2_D_3_, *n* = 249 and *n* = 383, and for 24-h Na^+^ and K^+^ excretion, *n* = 212 and *n* = 378, respectively. Glomerular filtration rate (GFR) was estimated by cystatin C based CKD-EPI formula. 25(OH)D_3_, 25-hydroxyvitamin D_3_; 1,25(OH)_2_D_3_, 1,25-dihydroxyvitamin D_3_; HDL, high-density lipoprotein; LDL, low-density lipoprotein.

Normally distributed variables were given as mean and standard deviation (SD), or as mean and 95% confidence intervals of the mean, and nonnormally distributed variables as median (25th to 75th percentile). Statistical significance was assumed at *P* less than 0.05. IBM Statistics SPSS version 29 was used (IBM SPSS, Armonk, New York, USA).

## RESULTS

### Study population and laboratory analyses

Demographics of the study population are presented in Table 1. The proportion of males and the mean age were similar in the groups with a positive and a negative family history of hypertension: 51.9 vs. 55.1% and 48.2 (12.7) vs. 48.1 (11.5) years, respectively. Additionally, body mass index, smoking status, alcohol consumption, and prevalence of diabetes did not differ between the groups. The prevalence of dyslipidaemia and the number of antihypertensive medication users, particularly those taking angiotensin receptor blockers and thiazide diuretics, were higher in the group with a family history of hypertension. Furthermore, in the group with a family history of hypertension, SBP and DBP were higher, whether measured by a physician in the office or by a research nurse in the laboratory (Table 1).

In comprehensive laboratory analyses, blood haemoglobin, plasma C-reactive protein, markers of renal function and the renin–angiotensin–aldosterone system, uric acid, plasma and urine sodium and potassium, vitamin D metabolites, parathyroid hormone, phosphate, urine albumin excretion, and the Cornell voltage-duration product were similar between the study groups (Table 2). In participants with a family history of hypertension, plasma total and low-density lipoprotein cholesterol were 0.16 (0.02–0.31; 95% confidence intervals) mmol/l higher, and calcium was 0.018 (0.01–0.034) mmol/l higher, with no differences in plasma triglycerides and high-density lipoprotein cholesterol, when compared to participants without a family history of hypertension (Table 2).

### Haemodynamic recordings

**FIGURE 1:**
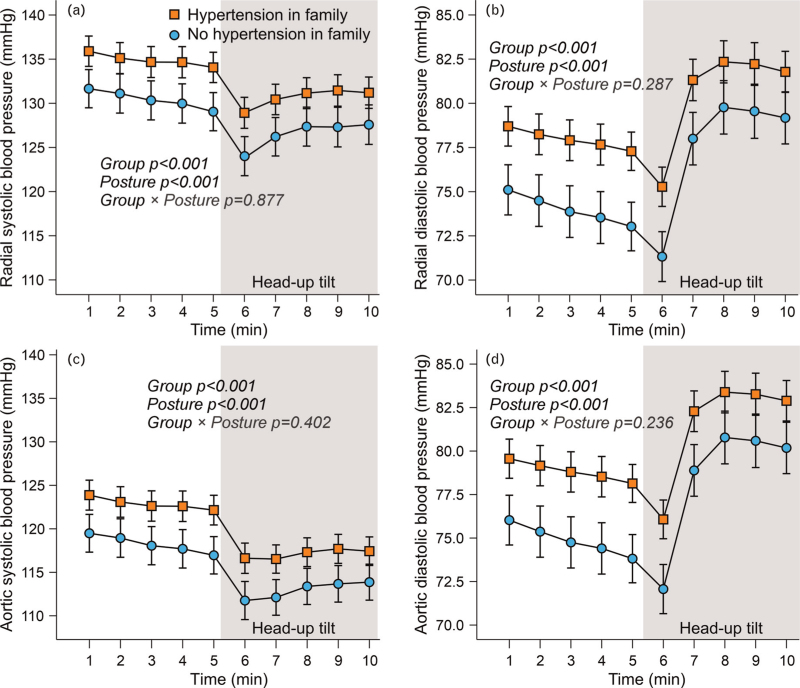
Radial (a) and aortic (c) systolic blood pressure, and radial (B) and aortic (D) diastolic blood pressure during passive head-up tilt in participants with and without hypertension in first-degree family members; mean and 95% confidence interval of the mean; statistics with generalized estimating equations.

**FIGURE 2:**
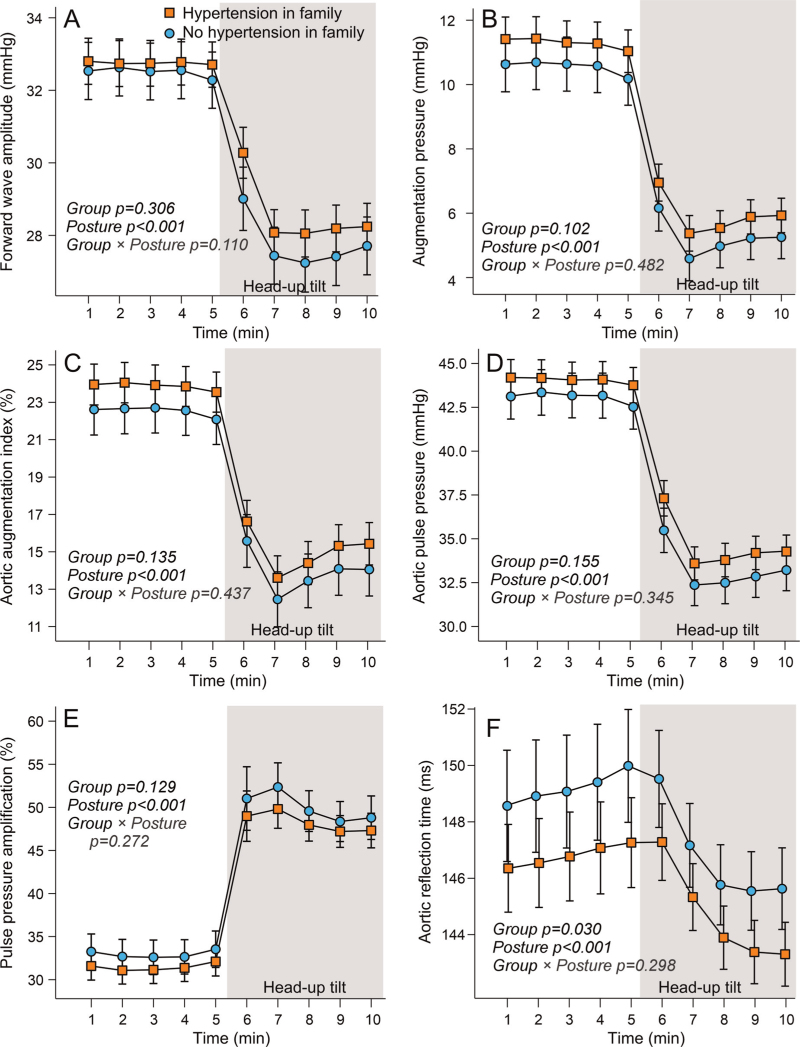
Forward wave amplitude (a), augmentation pressure (b), augmentation index (c), aortic pulse pressure (d), pulse pressure amplification (e), and aortic reflection time (f) during passive head-up tilt in participants with and without hypertension in first-degree family members; mean and 95% confidence interval of the mean; statistics with generalized estimating equations.

During the 10-min recordings, consisting of 5 min in the supine position and 5 min of head-up tilt, both radial and aortic SBP and DBPs were consistently higher in the group with a family history of hypertension (Fig. 1). No significant group × posture interactions were observed, that is, SBP decreased, and DBP increased equally during head-up tilt in both groups. FWA, augmentation pressure, aortic AIx, aortic pulse pressure, and pulse pressure amplification did not differ between the two groups in the supine or upright positions (Fig, 2). However, aortic reflection time was shorter in participants with a family history of hypertension. All variables shown in Fig. 2 decreased during head-up tilt, except for pulse pressure amplification, which increased; no significant differences were observed between the groups.

**FIGURE 3:**
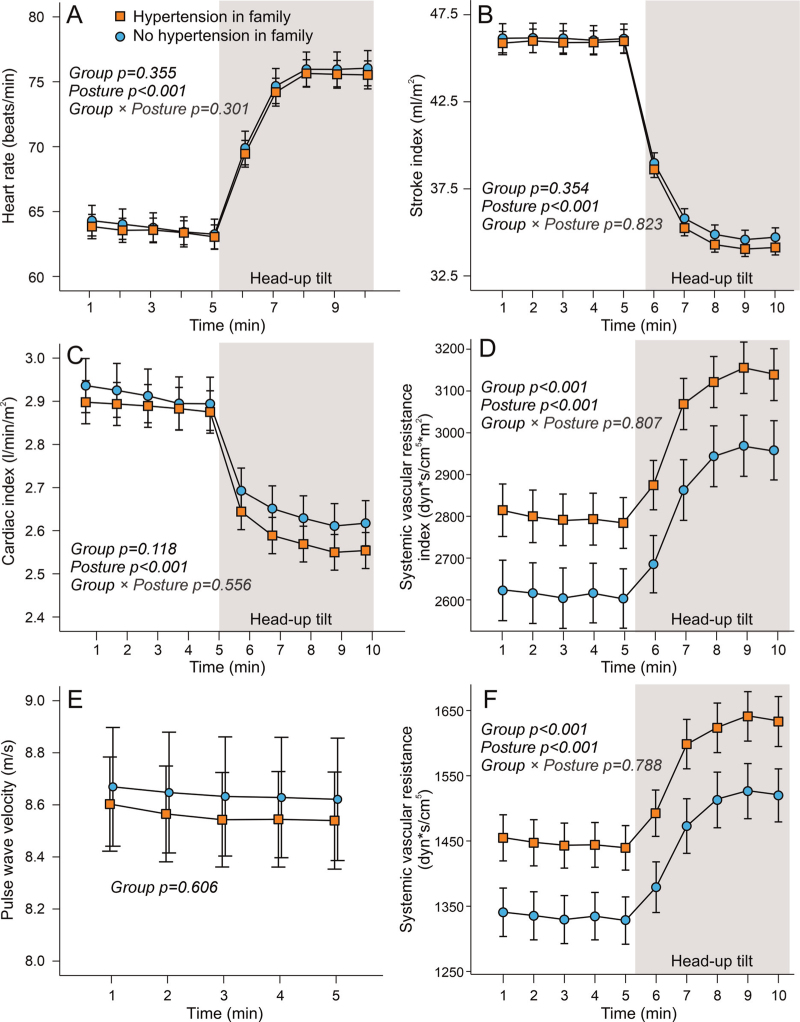
Heart rate (a), stroke index (b), cardiac index (c) systemic vascular resistance index (d), pulse wave velocity (e), and systemic vascular resistance (f) during passive head-up tilt in participants with and without hypertension in first-degree family members; mean and 95% confidence interval of the mean; statistics with generalized estimating equations.

Heart rate, stroke index, and cardiac index were similar in the two groups, regardless of body position (Fig. 3). In contrast, SVR and SVR index were higher in participants with a family history of hypertension than in controls. During head-up tilt, SVR index increased markedly compared with supine values, but no differences in the SVR increase between groups were observed (Fig. 3). Aortic-to-popliteal PWV was similar in the study groups (Fig. 3).

## DISCUSSION

In the present study, we investigated haemodynamics in individuals with and without a hypertensive first-degree family history. The results showed higher radial and aortic BP and higher SVR in participants with a known family history of hypertension. These results were consistent, as BP was higher in participants with a positive family history, regardless of whether it was measured by a physician, a research nurse, or tonometrically in the laboratory. These findings are in line with previous research, as a positive family history is an acknowledged risk factor for the development of hypertension [2,3].

BP is the product of cardiac output and SVR and is influenced by arterial stiffness [6]. In this study, higher BP in participants with a family history of hypertension appears to be driven primarily by elevated SVR, as cardiac output and arterial stiffness did not differ between the groups. Despite a higher prevalence of antihypertensive medication use, including angiotensin-receptor blockers and thiazide diuretics, which lower BP by reducing SVR, elevated BP and SVR were observed in participants with a family history of hypertension.

The aetiology of primary hypertension is multifactorial, with higher SVR being the hallmark of sustained hypertension [36,37]. A key factor in this pathophysiology is the reduction in lumen diameter of small arteries and arterioles, caused by structural vascular remodelling or increased vascular contraction. The latter is primarily mediated by the activation of the renin–angiotensin–aldosterone system, higher sympathetic tone, oxidative stress, immune activation, and endothelial dysfunction [38]. However, the extent to which the increase in SVR is genetically determined remains inconclusive. Although over 1400 single-nucleotide polymorphisms associated with hypertension have been identified, they explain less than one-third of the estimated heritability of hypertension and provide little mechanistic insight, as the vast majority of single-nucleotide polymorphisms are from noncoding regions of the genome, showing only pleiotropic associations [39]. In an experimental study, small blood vessels from individuals with a family history of hypertension exhibited increased sensitivity to vasoconstrictive substances compared with morphologically similar blood vessels from controls [40]. Additionally, individuals with a positive family history have shown enhanced microvascular reactivity to ischaemia, and impaired endothelium-mediated vasodilatation [41].

Among the potential haemodynamic contributors to elevated BP, arterial stiffness has been suggested to predict the development of hypertension [7,8] and to worsen prognosis [42]. Elevated SVR as a risk factor for incident hypertension has been less extensively studied. In a cohort of 73 men, baseline levels of this variable were not associated with future hypertension [43]. In a recent study of over 1200 participants, SVR was concluded to be the most significant predictive factor for incident hypertension [9]. However, this finding should be interpreted cautiously, as mean arterial pressure primarily determines SVR, and the conclusion may simply reflect that higher BP predicts higher future values [9]. The essential role of peripheral arterial resistance in sustained hypertension was evident in a study of 34 238 hypertensive individuals, where a combination of low or normal cardiac output and high vascular resistance was the most common haemodynamic phenotype across all grades of hypertension [37]. The relationship between arterial stiffness and hypertension is bidirectional; while hypertension increases arterial stiffness, stiffened arteries also elevate SBP [7]. Although SVR and arterial stiffness have rarely been evaluated together in studies on incident hypertension, from a pathophysiological standpoint, higher SVR would raise BP more in individuals with stiffer arteries, as wave reflection is related to both [44]. Additionally, in newly diagnosed hypertensive individuals, wave reflection and SVR have been identified as stronger determinants of pulse pressure amplification than arterial stiffness [45]. Notably, in our study, arterial stiffness did not differ between the groups.

A shorter aortic reflection time in individuals with a family history of hypertension, despite no differences in PWV, may appear contradictory. However, reflection time is influenced not only by large artery stiffness but also by reflection site distance and waveform shape [27], and the relationship between arterial stiffness and wave reflections is nonlinear [46]. Modelling studies indicate that peripheral resistance affects reflections indirectly by altering arterial pressure, which then impacts stiffness [46]. Nevertheless, our findings suggest that elevated SVR *in vivo* may still contribute to a shorter aortic reflection time. The graphs showing mean FWA, augmentation pressure, AIx, pulse pressure, and its amplification revealed nonsignificant trends toward less favourable values in individuals with a family history of hypertension. The present study may have been underpowered to detect differences in these variables.

Enhanced autonomic responses, such as hyperreactivity in a cold pressor test or an exaggerated orthostatic SBP response (i.e. higher upright than supine SBP), predict future hypertension [47,48]. In our study, autonomic activation was assessed using a head-up tilt test. Like during the supine measurements, participants with a family history of hypertension exhibited elevated BP and SVR also in the upright position. No additional differences in other haemodynamic parameters were observed. Thus, based on the present head-up tilt test results, major alterations in autonomic regulation probably do not account for the higher SVR and BP in individuals with a family history of hypertension.

Resistance vessels contract in response to an acute elevation in BP [49], while elevated SVR enhances pulse wave reflections [44]. In the present study, aortic reflection time was shorter in participants with a family history of hypertension, likely due to elevated SVR. However, the role of aortic reflection time as a risk factor for hypertension remains unclear. Large artery stiffness, aortic wave reflections, and SVR are all determinants of central BP [45,50]. Notably, the AIx did not differ between the two study groups, despite some reports have associated higher AIx with the risk of incident hypertension [7,9,10]. A possible explanation for this is the absence of differences in PWV and cardiac output between the study groups.

The groups compared in this study were relatively uniform. However, in addition to the higher prevalence of antihypertensive therapy, participants with a family history of hypertension also had a higher prevalence of dyslipidaemia, statin use, and elevated total and low-density lipoprotein cholesterol concentrations. Previous studies have identified an adverse lipid profile as a risk factor for incident hypertension [51,52]. Moreover, low-density lipoprotein cholesterol has been independently linked to BP through its effect on SVR, suggesting a connection between dyslipidaemia and the pathogenesis of primary hypertension [53]. In the present study, participants with a family history of hypertension had higher plasma total calcium levels than controls. Elevated plasma calcium levels have been associated with higher BP and SVR in earlier research [54]. However, the absolute plasma level differences of total and low-density lipoprotein cholesterol and calcium were quite small in our study.

The study has some limitations. The questionnaire did not specify the exact hypertensive family member. The noninvasive evaluation of haemodynamic parameters was based on mathematical analysis of bioimpedance and radial artery tonometric signals. However, radial pulse wave analysis was performed using a validated generalized transfer function [26], and the evaluation of cardiac stroke volume and PWV using impedance cardiography has been shown to correlate well with invasive measurements, three-dimensional ultrasound, and tonometric recordings of PWV [29,32,44]. In older participants, the current approach to evaluating FWA may be less precise than in younger individuals; however, the similar mean ages across study groups should help control for this potential confounding factor. Importantly, the beat-to-beat haemodynamic recording protocol provided continuous data from over 600 cardiac cycles, offering a significantly broader perspective on the haemodynamic system compared to most widely used tonometric recordings, which typically capture only 10–20 heartbeats. Although some criticism has been raised regarding the reliability of tonometric BP recordings [55], we recently reported that tonometric laboratory BP values well corresponded to the level of ambulatory daytime BP among 410 participants [56].

In conclusion, participants with hypertensive first-degree relatives exhibited higher central and peripheral BP, primarily driven by elevated SVR. This occurred without notable differences in large arterial stiffness or autonomic cardiovascular regulation. However, due to the cross-sectional design of this study, causality cannot be established. Altogether, these present findings highlight the role of SVR in the pathogenesis of primary hypertension.

## ACKNOWLEDGEMENTS

The authors sincerely thank our research nurses Emmi Hirvelä, Virpi Ryhänen, and Paula Liikanen for invaluable technical assistance. The authors wish to acknowledge CSC – IT Center for Science, Finland, for computational resources.

Data sharing statement: analyses and generated datasets that support the current study are not available publicly. The datasets are available from the corresponding author on reasonable request.

Novelty: the results have not been presented previously, in whole or in part.

Sources of support: this work was supported by Competitive State Research Financing of the Expert Responsibility Area of Tampere University Hospital (Grants 9AC076 and T63264); Finnish Foundation for Cardiovascular Research; Sigrid Jusélius Foundation; and Pirkanmaa Regional Fund of the Finnish Cultural Foundation.

### Conflicts of interest

There are no conflicts of interest.
